# HCV Infection Increases the Expression of ACE2 Receptor, Leading to Enhanced Entry of Both HCV and SARS-CoV-2 into Hepatocytes and a Coinfection State

**DOI:** 10.1128/spectrum.01150-22

**Published:** 2022-10-31

**Authors:** Tom Domovitz, Samer Ayoub, Michal Werbner, Joel Alter, Lee Izhaki Tavor, Yfat Yahalom-Ronen, Evgeny Tikhonov, Tomer Meirson, Yaakov Maman, Nir Paran, Tomer Israely, Moshe Dessau, Meital Gal-Tanamy

**Affiliations:** a Molecular Virology Lab, The Azrieli Faculty of Medicine, Bar-Ilan University, Safed, Israel; b The Laboratory of Structural Biology of Infectious Diseases, The Azrieli Faculty of Medicine, Bar-Ilan University, Safed, Israel; c Department of Infectious Diseases, Israel Institute for Biological Research, Ness Ziona, Israel; d The Lab of Genomic Instability and Cancer, The Azrieli Faculty of Medicine, Bar-Ilan University, Safed, Israel; e The Azrieli Faculty of Medicine, Bar-Ilan University, Safed, Israel; f Davidoff Cancer Center, Rabin Medical Center-Beilinson Hospital, Petah Tikva, Israel; University of Manitoba

**Keywords:** hepatitis C virus, SARS-CoV-2, coinfection, liver

## Abstract

Recent studies suggest the enhancement of liver injury in COVID-19 patients infected with Hepatitis C virus (HCV). Hepatocytes express low levels of angiotensin-converting enzyme 2 (ACE2), the SARS-CoV-2 entry receptor, raising the possibility of HCV-SARS-CoV-2 coinfection in the liver. This work aimed to explore whether HCV and SARS-CoV-2 coinfect hepatocytes and the interplay between these viruses. We demonstrate that SARS-CoV-2 coinfects HCV-infected Huh7.5 (Huh7.5^HCV^) cells. Both viruses replicated efficiently in the coinfected cells, with HCV replication enhanced in coinfected compared to HCV-mono-infected cells. Strikingly, Huh7.5^HCV^ cells were eight fold more susceptible to SARS-CoV-2 pseudoviruses than naive Huh7.5 cells, suggesting enhanced SARS-CoV-2 entry into HCV-preinfected hepatocytes. In addition, we observed increased binding of spike receptor-binding domain (RBD) protein to Huh7.5^HCV^ cells, as well as enhanced cell-to-cell fusion of Huh7.5^HCV^ cells with spike-expressing Huh7.5 cells. We explored the mechanism of enhanced SARS-CoV-2 entry and identified an increased ACE2 mRNA and protein levels in Huh7.5^HCV^ cells, primary hepatocytes, and in data from infected liver biopsies obtained from database. Importantly, higher expression of ACE2 increased HCV infection by enhancing its binding to the host cell, underscoring its role in the HCV life cycle as well. Transcriptome analysis revealed that shared host signaling pathways were induced in HCV-SARS-CoV-2 coinfection. This study revealed complex interactions between HCV and SARS-CoV-2 infections in hepatocytes, which may lead to the increased liver damage recently reported in HCV-positive COVID-19 patients.

**IMPORTANCE** Here, we provide the first experimental evidence for the coexistence of SARS-CoV-2 infection with HCV, and the interplay between them. The study revealed a complex relationship of enhancement between the two viruses, where HCV infection increased the expression of the SARS-CoV-2 entry receptor ACE2, thus facilitating SARS-CoV-2 entry, and potentially, also HCV entry. Thereafter, SARS-CoV-2 infection enhanced HCV replication in hepatocytes. This study may explain the aggravation of liver damage that was recently reported in COVID-19 patients with HCV coinfection and suggests preinfection with HCV as a risk factor for severe COVID-19. Moreover, it highlights the possible importance of HCV treatment for coinfected patients. In a broader view, these findings emphasize the importance of identifying coinfecting pathogens that increase the risk of SARS-CoV-2 infection and that may accelerate COVID-19-related co-morbidities.

## INTRODUCTION

Coronavirus (CoV) disease 2019 (COVID-19) caused by severe acute respiratory syndrome coronavirus 2 (SARS-CoV-2), has led to many deaths worldwide since its emergence in December 2019 in Wuhan, China. The disease symptoms range from mild to severe (1), that can progress to acute respiratory distress syndrome (ARDS) and death (2, 3). Epidemiological studies have associated factors such as older age, male sex, and comorbidities, including hypertension, heart disease, diabetes, and malignancy, with increased risk of severe COVID-19 (3). It is now evident that SARS-CoV-2 has broad organotropism, including the kidneys, liver, heart, and brain (4). Thereby, it can also contribute to multiorgan dysfunction such as acute cardiac injury, acute renal insufficiency, and liver damage, and can aggravate preexisting conditions (5). Understanding the effect of preinfection clinical status on SARS-CoV-2 infection outcomes is crucial for identification of the risk factors for severe illness, stratification of high-risk patients, prediction of clinical outcome and prioritizing and tailoring treatment regimens.

SARS-CoV-2 is an enveloped, non-segmented, positive-sense RNA virus. Its genome contains at least 6 open reading frames (ORFs), encoding structural and nonstructural proteins. The Spike (S) gene encodes the main envelope glycoprotein (6), which is a trimeric class I membrane fusion protein responsible for host-cell binding, fusion of the viral and host cell membranes and entry into the host cell. The receptor-binding domain (RBD) at the S1 subunit binds the angiotensin-converting enzyme 2 (ACE2) cellular receptor, triggering conformational changes of the S trimer (7, 8). Then, the S protein is cleaved at the S1/S2 by the cellular serine protease transmembrane serine protease 2 (TMPRSS2) at the cell surface or by cathepsin L in the endosomal compartment, thereby allowing fusion of viral and cellular membranes by the S2 subunit (9, 10). Therefore, expression of both ACE2 and TMPRSS2 determines the tissue tropism of SARS-CoV-2, and the course of the disease (11). Indeed, a broad spectrum of ACE2 and TMPRSS2 expression profiles was identified in lung, intestine, kidney, heart, adipose, reproductive tissues, brain, and liver tissues and cell lines (12, 13). It is highly important to identify factors affecting the expression of the SARS-CoV-2 entry determinants.

Liver tissue was found to express low levels of ACE2 and TMPRSS2 (12–17). Experiments with pseudoviruses and the authentic virus in various liver-derived cell lines and cellular models demonstrated their permissiveness to SARS-CoV-2 entry and replication (12, 14, 18). However, due to the clinical challenge of obtaining liver tissues during active SARS-CoV-2 infection, direct evidence for virus infection and replication in clinical samples from the liver is scarce. Recent studies identified SARS-CoV or SARS-CoV-2 RNA and coronavirus-like particles in liver samples obtained postmortem from patients who had severe COVID‐19-associated lung disease; abnormalities and fibrosis in the liver tissue were observed (19–24). Anomalies in liver biochemistry in COVID-19 patients is commonly reported, including elevations of serum alanine aminotransferase (ALT), aspartate aminotransferase (AST), serum bilirubin levels, and liver injury (25–30). Particularly, patients with preexisting liver disease showed higher risk of severe COVID-19, elevated liver enzymes levels and higher mortality rate, although the reports are inconsistent (31–36). Still, it remains to be determined whether liver damage is contributed by direct SARS-CoV-2 infection or is an indirect consequence of pathological processes, such as inflammation, occurring during COVID-19 (37).

Hepatitis C virus (HCV) infects 1–2% of the world's population and can lead to chronic liver disease, liver cirrhosis and hepatocellular carcinoma, cumulatively posing a major global health burden (38). HCV primarily infects hepatocytes (39), and, therefore, HCV- SARS-CoV-2 coinfection in the liver may be possible. However, it is currently unknown whether preexisting infection with HCV impacts the susceptibility of the liver to SARS-CoV-2 infection. Only limited data are available on liver pathology and mortality rates in COVID-19 patients with chronic HCV infection. Butt et al. reported on a higher percentage of overall hospitalization of HCV-infected COVID-19 patients, but not on a higher mortality rate, compared to COVID-19 patients without HCV (40). Other studies showed higher prevalence of hepatic manifestations, liver damage, liver enzymes levels, and mortality rate among COVID-19-HBV and COVID-19-HCV coinfected patients compared to COVID-19 patients (40, 41). Although the interplay between HCV and SARS-CoV-2 infections is not known, studies suggested that SARS infection in chronic HCV and HBV patients may cause severe hepatitis due to higher HBV/HCV replication (42). Moreover, preinfection with HBV prolonged the clearance of SARS-CoV-2 infection (43). Taken together, the data reported thus far suggest a complex interaction between chronic HCV infection and SARS-CoV-2 infection. Moreover they suggest that treatment of COVID-19-HCV coinfected patients with direct-acting antivirals (DAAs) for HCV cure may improve disease severity and clinical outcome of COVID-19. Here, we show for the first time that both viruses coinfect liver cells, and show enhanced replication and infection in coinfected hepatocytes.

## RESULTS

### HCV and SARS-CoV-2 coinfect and co-replicate in hepatocytes.

**FIG 1:**
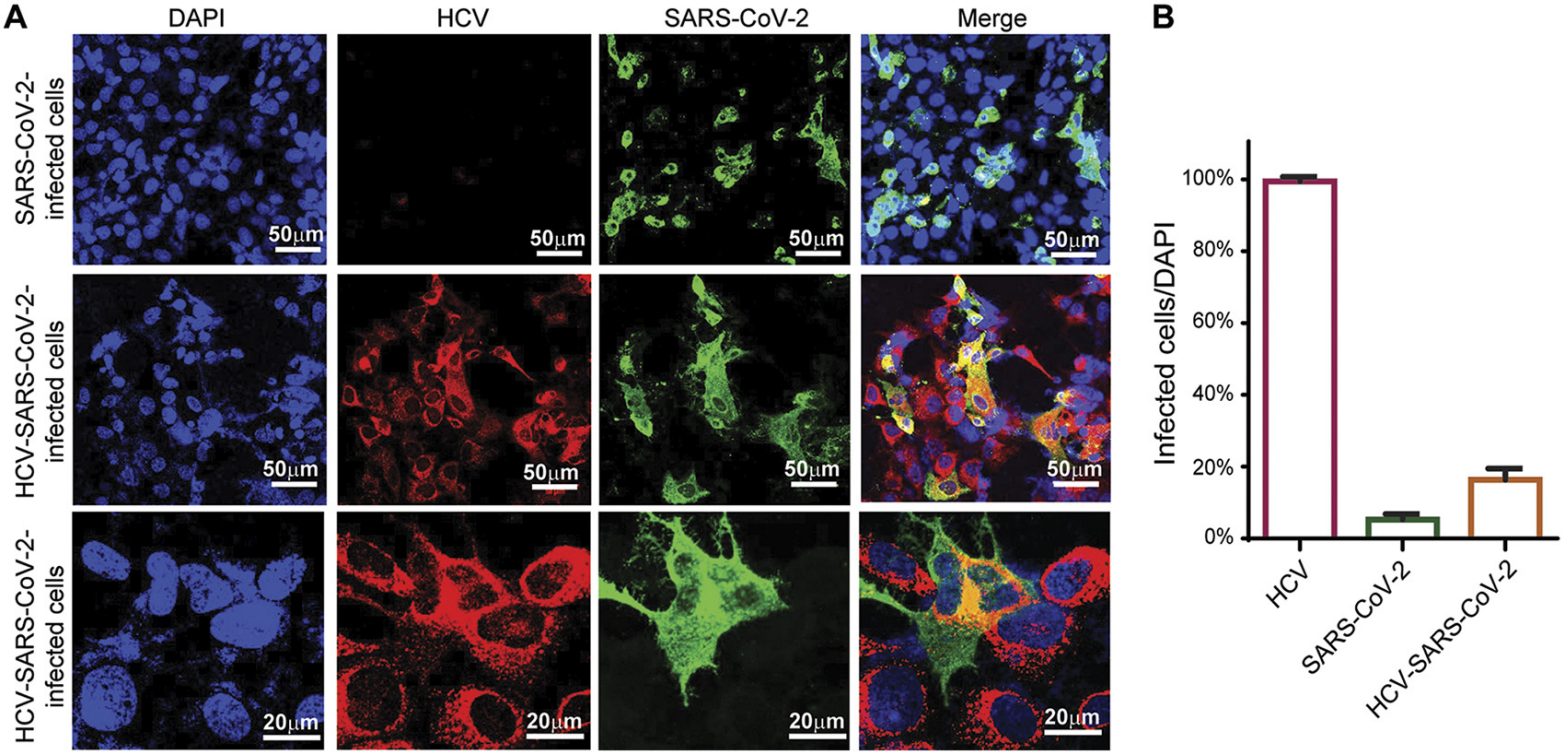
HCV and SARS-CoV-2 coinfect hepatocytes. (A) Evaluation of HCV and SARS-CoV-2 coinfection in Huh7.5 cells by immunostaining. Non-infected Huh7.5 (top panel) and Huh7.5^HCV^ cells (middle and bottom panels) were exposed to SARS-CoV-2 for 48 h and then fixed and co-stained with HCV-positive serum and rabbit anti-SARS-CoV-2 serum, followed by anti-human cy3, and Alexa Fluor 488 conjugated goat anti-rabbit secondary antibodies, respectively. Images were visualized by fluorescence microscopy. Scale bars: 50 μm/20 μm. (B) Quantification of the percent of HCV/SARS-CoV-2/HCV- SARS-CoV-2 coinfection, calculate from at least 6 images for each.

Previous studies demonstrated that the human hepatoma cell line Huh7.5, which supports HCV replication *in vitro*, is susceptible to infection by SARS-CoV-2 and other coronaviruses. In addition, this cell line was recently employed to identify host pathways that are important for the SARS-CoV-2 life cycle (14). Here, this cell line was used to explore the interplay between HCV and SARS-CoV-2 infections. First, we aimed to evaluate whether HCV and SARS-CoV-2 coinfection occurs in hepatocytes. Following a 48 h exposure of HCV-preinfected and noninfected control Huh7.5 cells to SARS-CoV-2, cells were co-immunostained to determine the coinfection rate. Indeed, cells staining for both HCV and SARS-CoV-2 were identified. Their morphology was similar to that of cells infected with HCV alone, and indicated no cytopathic effect up to 72 h postinfection (Fig. 1A), which aligns with a previous report showing limited cell death in SARS-CoV-2-infected Huh7.5.1 cells (14). Higher resolution images of the HCV-SARS-CoV-2-coinfected cells clearly showed regions of co-localization of proteins from both viruses, as well as regions with proteins from only one of the viruses (Fig. 1A, lower panels). Higher number of SARS-CoV-2 infected cells were observed in the coinfected culture than in SARS-CoV-2 infection alone (Fig. 1B).

**FIG 2:**
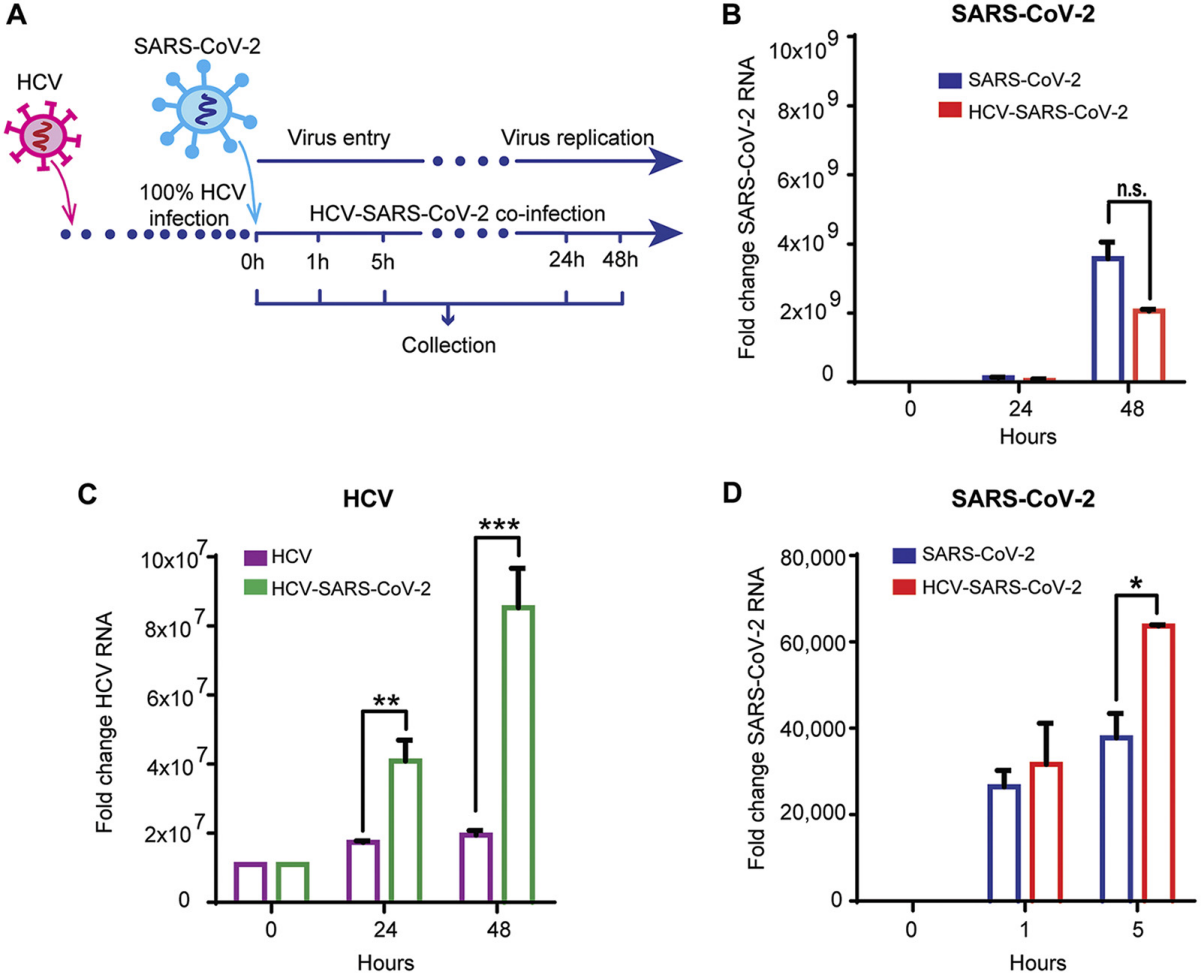
HCV and SARS-CoV-2 co-replicate in hepatocytes. (A) Experimental setup for HCV-SARS-CoV-2 coinfection. Non-infected Huh7.5 and Huh7.5^HCV^ cells were co-infected with SARS-CoV-2 at MOI of 0.01, and harvested 0, 1, 5, 24 and 48 h postinfection. RNA was extracted and qRT-PCR was performed to determine SARS-CoV-2 RNA level (B) and HCV RNA level (C) 0, 24 and 48 h after SARS-CoV-2 infection, and SARS-CoV-2 RNA levels (D) at 0, 1, and 5 h postinfection. Differential expression was calculated, with GAPDH as an endogenous control. Fold change was calculated compared to Huh7.5 control cells. Mean fold changes are shown ± standard deviation (SD), from 3 independent experiments (*P = 0.0229, **P = 0.0026, ***P = 0.0006, *t* test).

To determine whether the two viruses co-replicate in hepatocytes, the dynamics of RNA levels of the two viruses were monitored over time after infection (Fig. 2A). An overall increase in RNA levels of both viruses was detected over the 48 h after SARS-CoV-2 infection, suggesting efficient replication of both viruses in the coinfected cells (Fig. 2B and C). The increase in SARS-CoV-2 RNA levels was slightly smaller in the coinfected compared to SARS-CoV-2 mono-infected cells; however, this effect was not statistically significant (Fig. 2B). In parallel, while HCV RNA levels remained stable over time in the Huh7.5^HCV^ cells, they increased in the coinfected cells, suggesting that the presence of SARS-CoV-2 enhanced HCV RNA replication (Fig. 2C). We then aimed to determine the level of SARS-CoV-2 RNA at early time points postinfection. Interestingly, 5 h postinfection, higher levels of SARS-CoV-2 RNA were observed in the coinfected cells compared to control cells infected with SARS-CoV-2 only (Fig. 2D). Overall, these observations suggest efficient coinfection and co-replication of HCV and SARS-CoV-2 in human hepatocytes.

### HCV infection enhances SARS-CoV-2 entry into hepatocytes.

**FIG 3:**
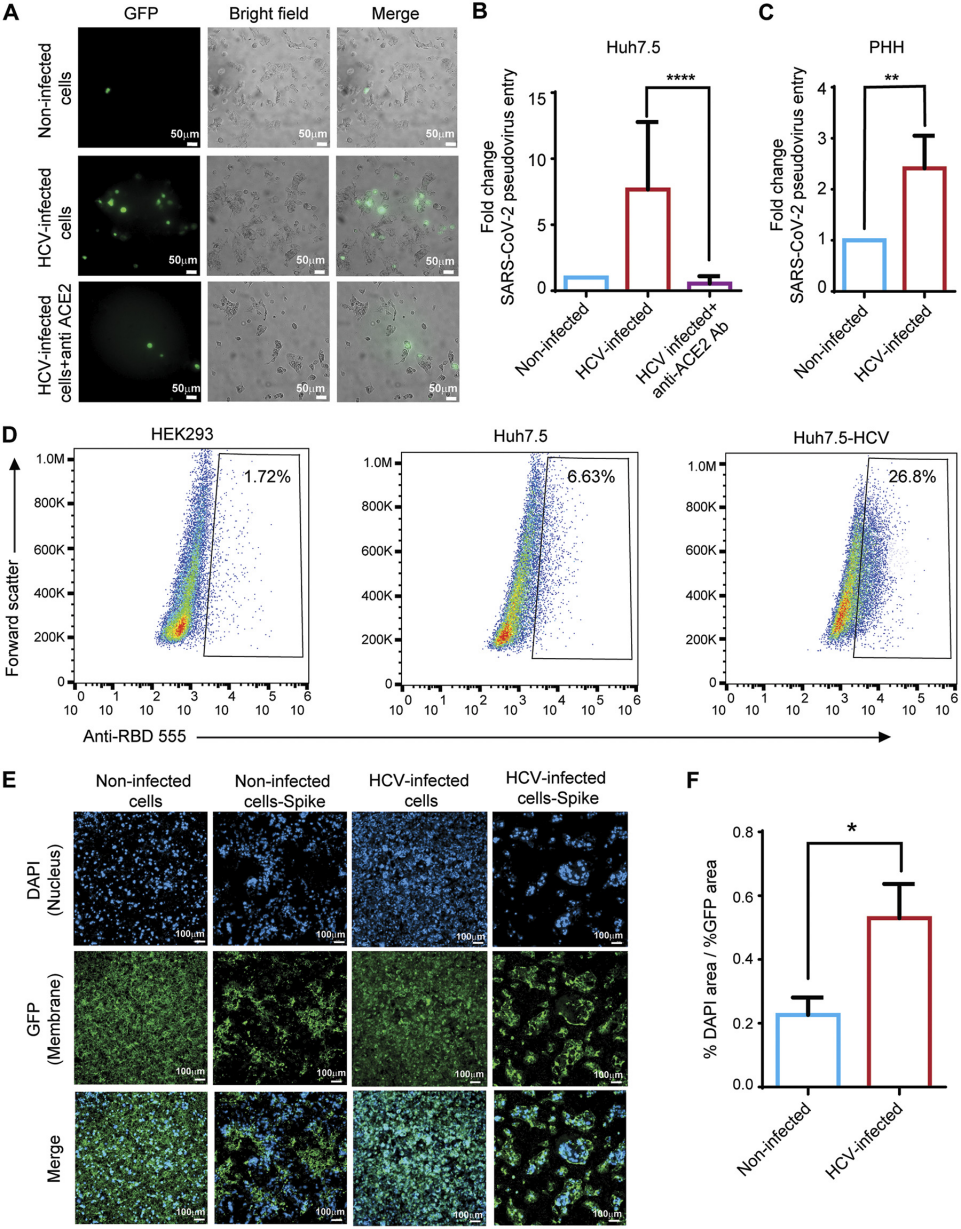
HCV infection enhances SARS-CoV-2 entry into hepatocytes. (A) Non-infected Huh7.5 and Huh7.5^HCV^ cells were incubated overnight with SARS-CoV-2-spike pseudoparticles with or without an anti-ACE2 antibody. Samples were visualized by fluorescence microscopy. Scale bars: 50 μm. (B) After a 24 h of incubation, all cells and GFP-positive cells were counted by fluorescence microscopy, and fold change of the number of Huh7.5^HCV^ GFP-positive cells was calculated compared to the number of Huh7.5 GFP-positive cells (****P < 0.0001, *t* test). (C) Non-infected and HCV-infected PHH were incubated overnight with SARS-CoV-2-spike pseudoparticles. Samples were visualized by fluorescence microscopy. GFP-positive cells were counted by fluorescence microscopy, and fold change of the number of GFP-positive cells was calculated (**P = 0.0015, *t* test). (D) RBD was incubated with HEK293, non-infected Huh7.5, and Huh7.5^HCV^ cells and then anti-RBD 555 was added. Flow cytometry was performed to evaluate RBD binding to the cells. (E) Non-infected Huh7.5 and Huh7.5^HCV^ cells were transfected with a spike-encoding plasmid. Following a 24-h incubation, the cells were stained with the CellMask Deep Red Plasma Membrane Stain and DAPI nuclear stain. Samples were visualized by fluorescence microscopy. Scale bars: 100 μm. (F) Percentage of DAPI staining relative to the percentage of membrane staining was quantified for Huh7.5 non-infected-spike cells compared to Huh7.5^HCV^ -spike cells (*P = 0.0119, *t* test).

At early time points postinfection, SARS-CoV-2 RNA levels may reflect the quantity of viruses that entered the cells. Therefore, the higher SARS-CoV-2 RNA level detected 5 h after infection of HCV-preinfected cells suggests enhanced entry of SARS-CoV-2 into these cells. To explore this possibility, we compared the uptake of GFP-reporter pseudoparticles bearing SARS-CoV-2-spike by Huh7.5^HCV^ versus Huh7.5 cells. Fig. 3A and B show that HCV-preinfection increased cell susceptibility to SARS-CoV-2-spike pseudoparticle infection, by approximately eight fold. To further assess the dependence of the enhanced entry of SARS-CoV-2-spike pseudoparticles into Huh7.5^HCV^ on interaction between the SARS-CoV-2 spike and the ACE2 receptor, the experiment was repeated in the presence of an anti-ACE2 antibody. As expected, anti-ACE2 antibody treatment abolished entirely the entry of SARS-CoV-2-spike pseudoparticles to the cells (Fig. 3B). We also validated the enhanced entry of SARS-CoV-2-spike pseudoparticles in HCV-infected primary human hepatocytes (PHH) (50% infected with HCV) (Fig. 3C).

Next, we measured the effect of HCV infection on each of the two SARS-CoV-2 entry steps: spike RBD binding to the ACE2 receptor and spike cleavage by cellular proteases like TMPRSS2. Flow cytometry analyses found no significant RBD binding to HEK293 cells that do not express ACE2 (1.72% positive cells), and low levels of RBD binding to Huh7.5 cells expressing low levels of ACE2 (6.63% positive cells). In contrast, higher levels of RBD binding were detected (26.8% positive cells) in Huh7.5^HCV^ cells, suggesting that higher levels of ACE2 are expressed on HCV-infected cells, leading to increased ACE2-RBD binding events (Fig. 3D). To further characterize these effects on spike-mediated membrane fusion, we performed fusion assay by overexpression of spike in Huh7.5^HCV^ and Huh7.5 cells. In this assay, cell-cell membrane fusion will only occur between cells that express spike and ACE2 receptor on their surfaces. While no cell-cell fusion was observed in Huh7.5 control cells (Fig. 3E), cell-cell fusion events were detected in non-infected, spike-expressing Huh7.5, and significantly increased in spike-expressing Huh7.5^HCV^ cells (Fig. 3E and F). In addition, Huh7.5^HCV^-spike cells fused into big syncytia, each containing multiple nuclei (Fig. 3E). Taken together, these observations show that the two spike-mediated viral entry steps are enhanced in the presence of HCV.

### HCV infection induces increased expression of SARS-Cov-2 entry factors in hepatocytes.

**FIG 4:**
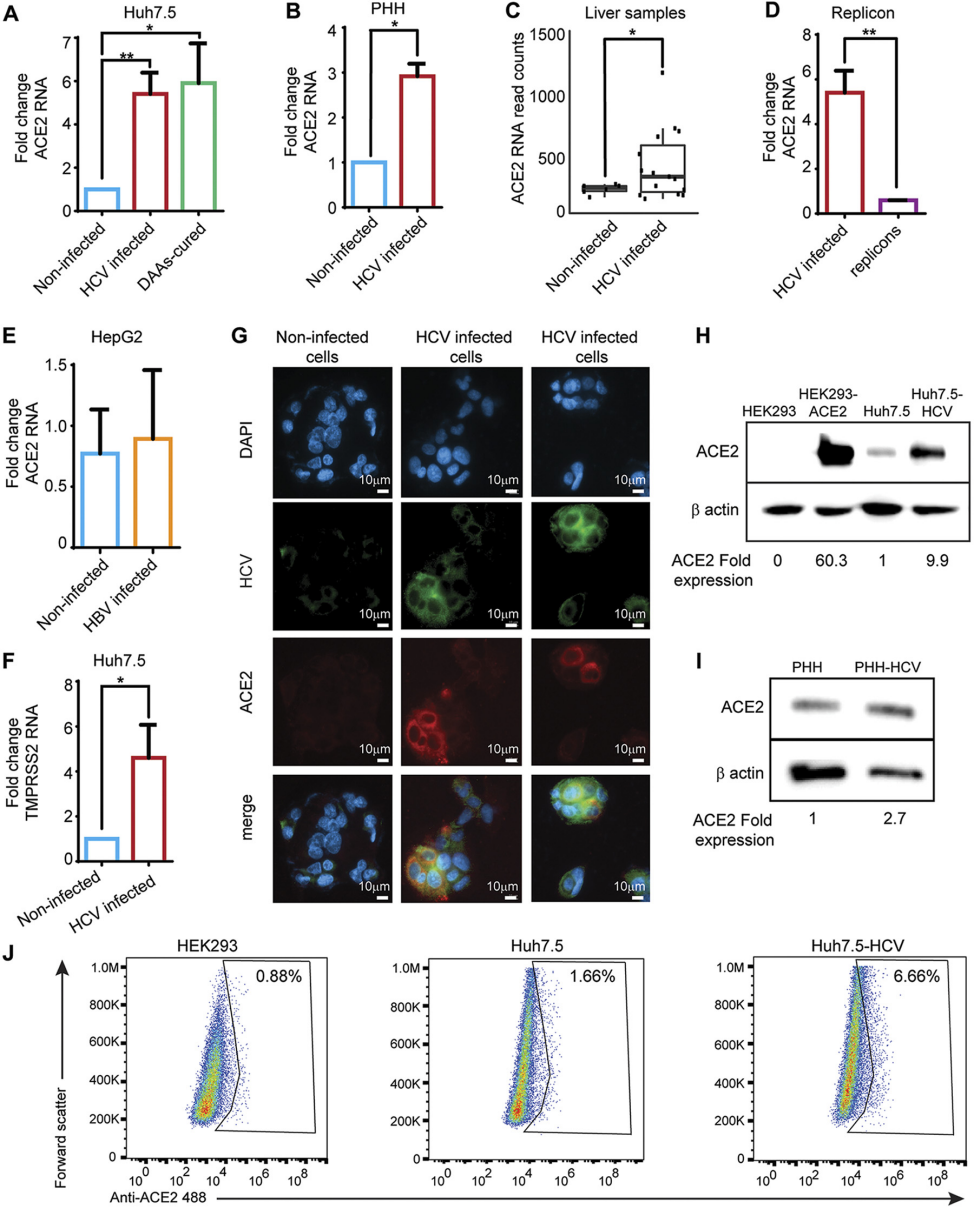
HCV infection upregulates ACE2 expression in hepatocytes. (A) qRT-PCR quantification of ACE2 mRNA levels in non-infected Huh7.5 cells, Huh7.5^HCV^ cells, and HCV-cured cells. Fold change was calculated compared to Huh7.5 control cells. Mean fold changes are shown ± SD, from three independent experiments (*P = 0.0149, **P = 0.0015, *t* test). (B) Quantification of ACE2 mRNA levels in non-infected PHH cells compared to HCV-infected PHH cells, as described above in (A). Mean fold changes are shown ± SD, from 3 independent experiments (*P = 0.0104, *t* test). (C) Shown are distributions of normalized read counts of ACE2 in normal (*n* = 6) and HCV-infected (*n* = 15) human livers (low interferon-stimulated genes) (*P* < 0.021, *t* test). (D) Quantification of ACE2 mRNA levels in Huh7.5^HCV^ cells compared to replicons cells, performed as described in (A). Mean fold changes are shown ± SD, from 3 independent experiments (**P = 0.0072, *t* test). (E) Quantification of ACE2 mRNA levels in HepG2 cells compared to HBV-infected HepG2 cells, performed as described in (A). (F) Quantification of TMPRSS2 RNA levels in non-infected Huh7.5 cells compared to Huh7.5^HCV^ cells, performed as described in (A). Mean fold changes are shown ± SD, from three independent experiments (*P = 0.0131, *t* test). (G) Immunostaining for ACE2 protein expression in non-infected Huh7.5 cells and Huh7.5 cell culture sample 50% infected with HCV. The cells were stained with HCV-positive serum and anti-human 488 Alexa fluor antibodies to detect HCV infection, and with anti-ACE2 and anti-rabbit Cy3 antibodies to detect ACE2 protein. Scale bars: 10 μm. (H) Lysates of HEK293 cells, HEK293 cells stably expressing ACE2, non-infected Huh7.5 cells, and Huh7.5^HCV^ cells were analyzed by Western blot using antibodies for ACE2 and β-actin. (I) Lysates of non-infected PHH cells and HCV-infected PHH cells were analyzed by Western blot using antibodies for ACE2 and β-actin. (J) HEK293, non-infected Huh7.5 and Huh7.5^HCV^-infected cells were incubated with anti-ACE2 488. Flow cytometry was performed to evaluate ACE2 level on the cells.

We next aimed to further explore the mechanism of HCV-mediated enhanced SARS-CoV-2 entry into hepatocytes. In line with the suggested higher ACE2 expression in Huh7.5^HCV^ cells (Fig. 3), we found approximately five fold higher ACE2 receptor RNA levels in Huh7.5^HCV^ compared to non-infected Huh7.5 cells (Fig. 4A). Importantly, increased ACE2 expression remains persistent also in HCV-cured cells following treatment with DAAs (Fig. 4A). Increased ACE2 mRNA expression was also measured in HCV-infected PHH (Fig. 4B), and in HCV-infected (low interferon-stimulated genes) liver samples compared to normal liver samples obtained from the NCBI Gene Expression Omnibus (GSE84346) (Fig. 4C). However, increased expression of ACE2 mRNA was not recapitulated in a cell line expressing the HCV-N subgenomic replicons that include only HCV nonstructural proteins (NS3, NS4A, NS4B, NS5A, and NS5B) (Fig. 4D), suggesting that the increased expression of ACE2 may be mediated by HCV structural proteins. ACE2 mRNA expression was also unchanged in HepG2 cells infected with HBV lentivirus expressing HBV proteins (Fig. 4E), pointing to an HCV-specific effect. In addition, the RNA level of the cellular serine protease TMPRSS2 were elevated in Huh7.5^HCV^ cells (Fig. 4F).

To determine whether HCV infection increases ACE2 protein expression, non-infected Huh7.5 cells and a Huh7.5 cell cultures in which 50% were infected with HCV, were immunostained with specific antibodies for the detection of HCV proteins and ACE2. No staining was detected in control cells (Fig. 4G, upper panel) and the non-infected cells neighboring HCV-infected cells (Fig. 4G, lower panel), likely due to the low level of ACE2 expression in Huh7.5 cells. In contrast, co-immunostaining was observed in the HCV-infected Huh7.5 cells (Fig. 4G, lower panel), suggesting that the control over increased ACE2 expression is cell autonomous and specific to HCV-infected cells. Western blot analysis supports these results, indicating that the ACE2 protein levels are not detected in HEK293 cells, are high in HEK293-ACE2 cells (HEK293 stably expressing hACE2), low in Huh7.5 cells, and significantly increased in Huh7.5^HCV^ cells (Fig. 4H). Similar differences were observed in non-infected versus HCV-infected PHH (Fig. 4I). We also validated that expression level of ACE2 is upregulated on Huh7.5^HCV^ cells by flow cytometry (Fig. 4J). Collectively, these data indicate that HCV infection induces expression of ACE2 on hepatocytes, which may underlie enhanced SARS-CoV-2 entry into HCV-infected hepatocytes.

### ACE2 expression affects HCV infection by enhancing viral entry into hepatocytes.

**FIG 5:**
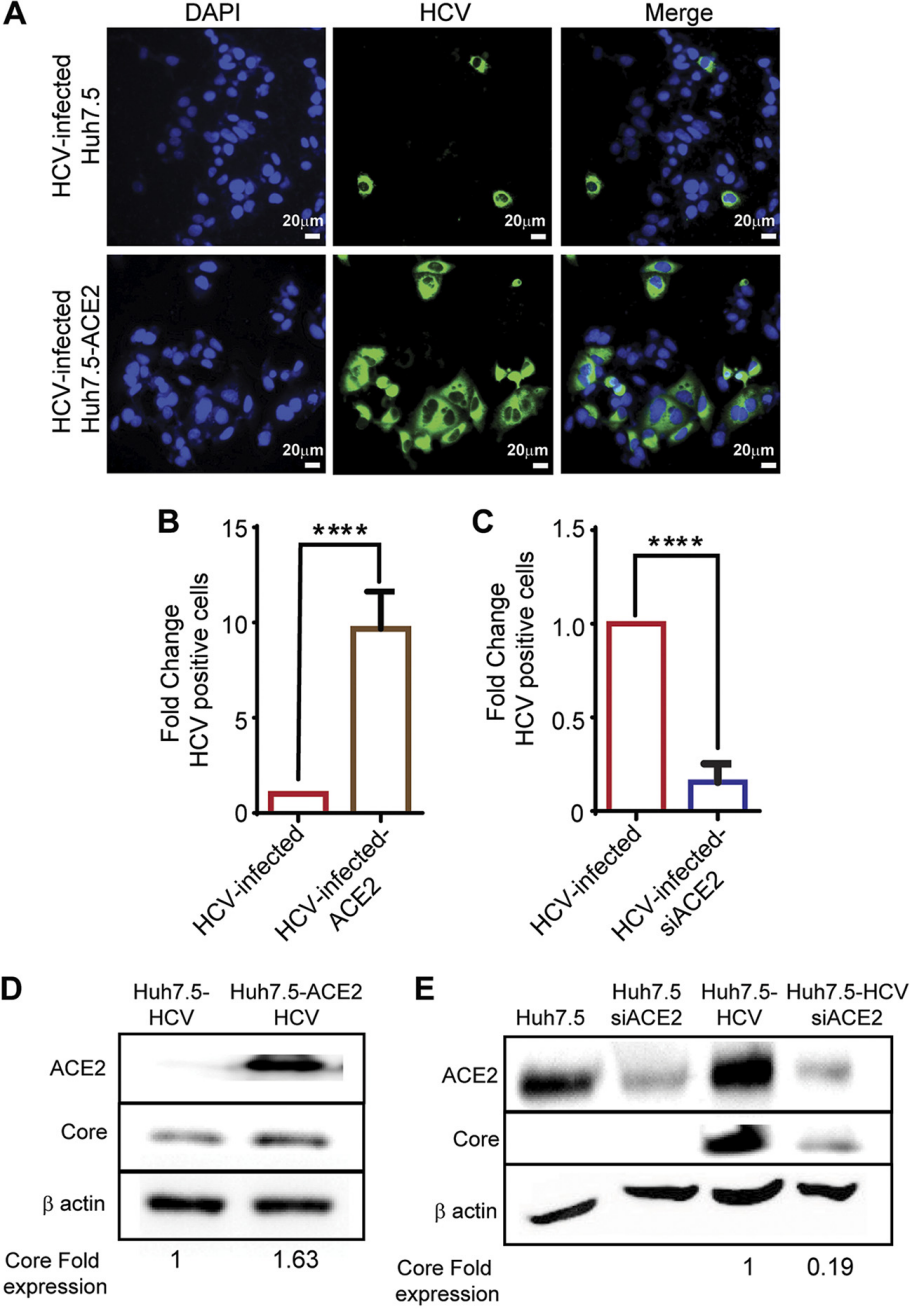
ACE2 expression increases HCV infection. (A) Huh7.5 cells were transfected with an ACE2-encoding plasmid, and 48 h after transfection, ACE2-overexpressing cells and control cells were infected with HCV. At 72 h postinfection, cells were fixated and stained with HCV-positive serum, anti-human 488 Alexa fluor antibodies, to detect HCV infection, and with DAPI nuclear stain. Scale bars: 20 μm. (B) Numbers of HCV-infected Huh7.5 and ACE2-overexpressing HCV-infected Huh7.5 cells were determined, and percentage of infection was calculated and is presented as fold change from control. Mean fold changes are shown ± SD, from three independent experiments (****P < 0.0001, *t* test). (C) Huh7.5 cells were transfected with ACE2 siRNA, and 48 h after, were infected with HCV. At 72 h postinfection, cells were fixated and stained with HCV-positive serum, anti-human 488 Alexa fluor antibodies, to detect HCV infection, and with DAPI. Number of HCV-infected cells were quantified, and percentage of infection was calculated and is presented as fold change from control. Mean fold changes are shown ± SD, from 3 independent experiments (****P < 0.0001, *t* test). (D) Huh7.5 cells and Huh7.5 cells were transfected with an ACE2-encoding plasmid; 48 h after transfection cells were infected with HCV. At 72 h postinfection, cell lysates were analyzed by Western blot using antibodies for Core, ACE2 and β-actin. (E) Huh7.5 cells were transfected with ACE2 siRNA for 24 h, and then infected with HCV. Cell lysates were analyzed by Western blot using antibodies for Core, ACE2 and β-actin.

In light of the observed upregulation of ACE2 expression in HCV-infected cells, we postulated that ACE2 plays a role in the HCV life cycle. To examine the effect of ACE2 protein levels on HCV infection, ACE2 was either overexpressed or knocked down in Huh7.5 cells (Huh7.5-ACE2/Huh7.5-siACE2), and then the cells were infected with HCV. Immunostaining for HCV proteins 72 h postinfection uncovered a higher percentage of HCV infection in Huh7.5-ACE2 cells (Fig. 5A, lower panel) compared to control Huh7.5 cells (Fig. 5A, upper panel). Infection rates were approximately five-fold higher and five-fold lower in Huh7.5-ACE2 (Fig. 5B) and Huh7.5-siACE2 cells (Fig. 5C), respectively. Western blot analysis showing a higher level of HCV core protein in Huh7.5-ACE2 (Fig. 5D), versus a lower level of HCV core protein in Huh7.5-siACE2 cells (Fig. 5E) compared to control, verified these results.

**FIG 6:**
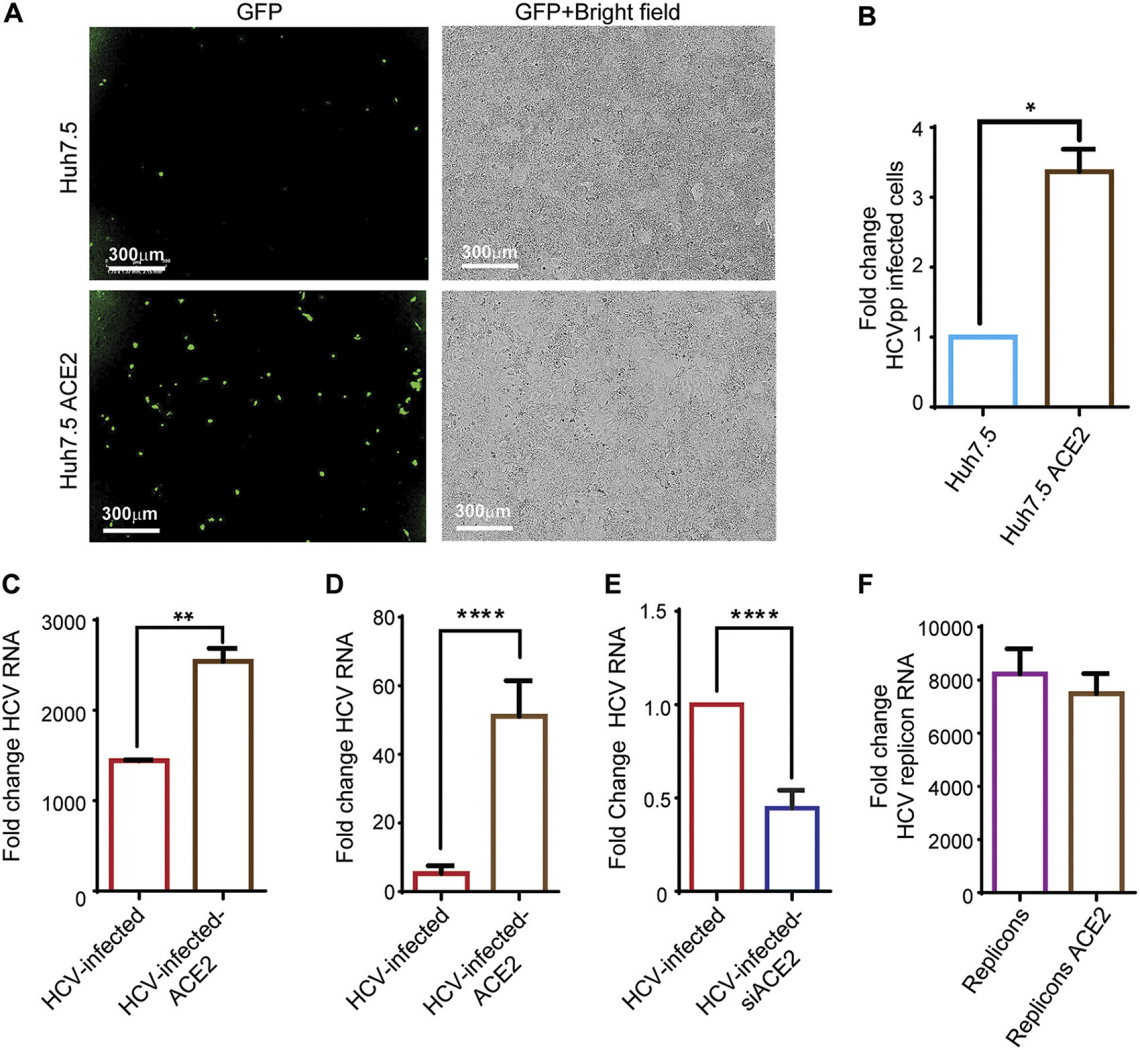
ACE2 expression enhances HCV entry. (A) Huh7.5 cells were transfected with an ACE2-encoding plasmid; 48 h following transfection, HCVpp were added to the transfected and non-transfected cells and cells were monitored using IncuCyte. Scale bars: 300 μm. (B) Following 24 h from HCVpp addition, all cells and GFP-positive cells were counted by fluorescence microscopy, and fold change was calculated compared to the number of non-transfected GFP-positive cells. Mean fold changes are shown ± SD, from 3 independent experiments (*P = 0.0299, *t* test). (C) Huh7.5 cells and ACE2-overexpressing Huh7.5 cells were mixed with HCV for 1 h in 4°C. RNA levels of cell-bound HCV were determined by qRT-PCR. Fold change was calculated compared to non-infected Huh7.5 cells. Mean fold changes are shown ± SD, from three independent experiments (**P = 0.0087, *t* test). (D) Huh7.5 cells were transfected with an ACE2-encoding plasmid; 48 h after transfection ACE2-overexpressing cells and control cells were infected with HCV. Thereafter (72 h), HCV RNA levels were quantified by qRT-PCR. Fold change was calculated compared to non-infected Huh7.5 cells. Mean fold changes are shown ± SD, from 3 independent experiments (***P = <0.0001, *t* test). (E) Huh7.5 cells were transfected with ACE2 siRNA; 48 h after transfection, siACE2-overexpressing cells and control cells were infected with HCV. At 72 h postinfection, HCV RNA levels were quantified by qRT-PCR, as described above. Mean fold changes are shown ± SD, from 3 independent experiments (*P = 0.0115, *t* test). (F) Replicon cells were transfected with ACE2 plasmid. At 48 h posttransfection, RNA was extracted, and replicon RNA levels were quantified by qRT-PCR in replicon and ACE2-overexpressing replicon cells and compared to control Huh7.5 cells. Mean fold changes are shown ± SD, from 3 independent experiments.

To determine whether ACE2 impacts HCV infection via modulation of HCV entry, GFP-reporter pseudoparticles bearing HCV E1E2 envelope proteins (HCVpp) were incubated with Huh7.5 and Huh7.5-ACE2 cells. A more than 3-fold increase in HCVpp uptake was observed in Huh7.5-ACE2 cells compared to control Huh7.5 (Fig. 6A and B), demonstrating that ACE2 plays a role in HCV entry. In parallel, incubation of the cells with HCV at 4°C for 1 h, which allows for virus binding only, without viral entry, resulted in approximately two-fold higher viral RNA in Huh7.5-ACE2 cells compared to Huh7.5 cells, suggesting a role for ACE2 in HCV binding to its host (Fig. 6C).

To evaluate whether ACE2 expression also has an effect on HCV RNA replication, HCV RNA levels in Huh7.5-ACE2, Huh7.5-siACE2, and control Huh7.5 cells were quantitated 72 h postinfection, as well as in HCV replicon cells with or without overexpression of ACE2. While HCV RNA levels were higher in Huh7.5-ACE2 (Fig. 6D) and lower in Huh7.5-siACE2 cells (Fig. 6E) compared to control cells, they were similar in replicon cells with versus without ACE2 overexpression (Fig. 6F). In conclusion, ACE2 increases viral entry into Huh7.5-ACE2 cells without affecting HCV RNA replication.

### Transcriptome analysis reveals shared pathways in HCV/SARS-CoV-2 mono versus coinfections.

**FIG 7:**
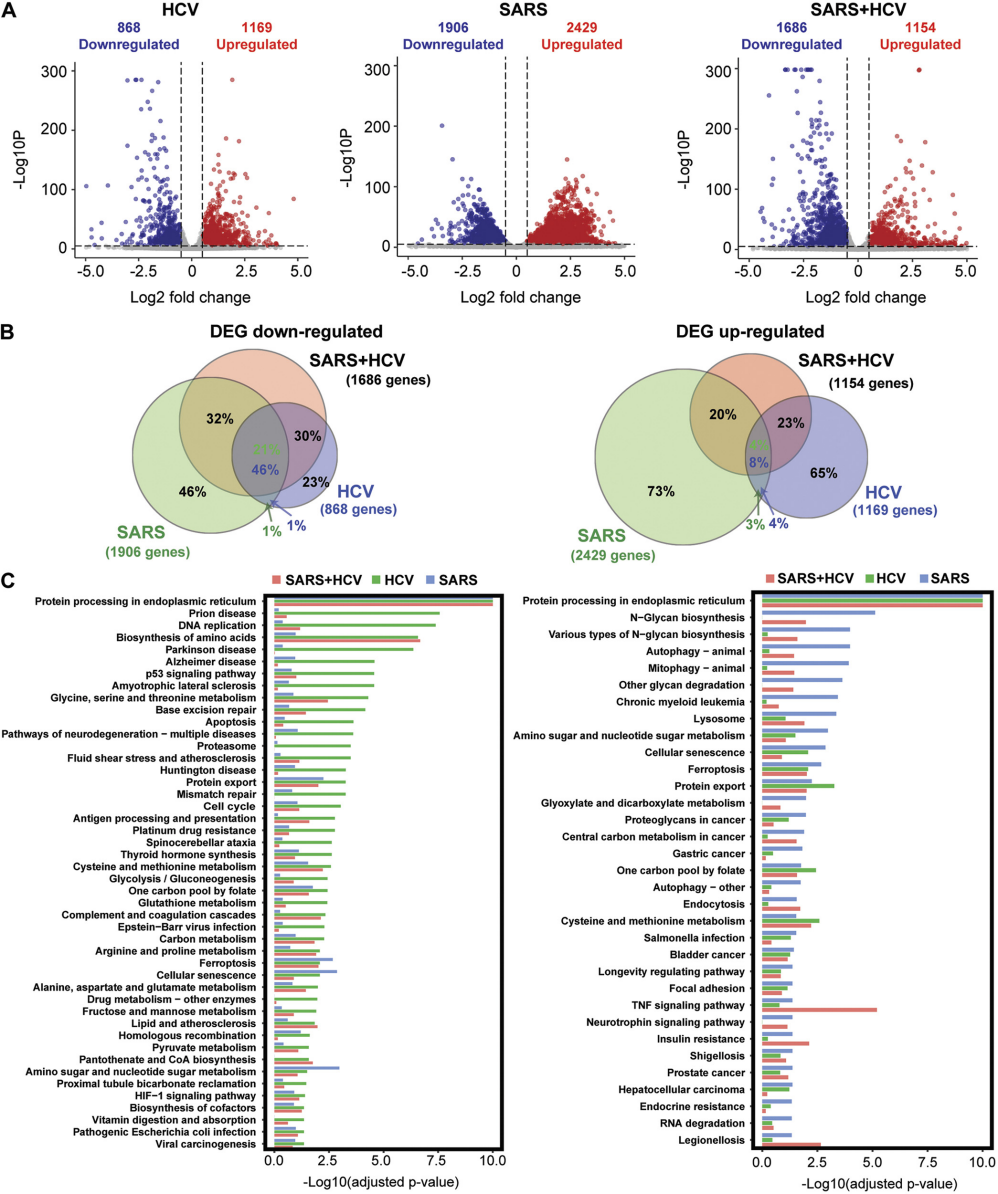
Differentially expressed genes and pathway analysis for HCV/SARS-CoV-2 mono- and coinfections. (A) Volcano plot showing upregulated (red) and downregulated (blue) genes in HCV-infected, SARS-CoV-2-infected and HCV-SARS-Cov-2 coinfected Huh7.5 cells compared to non-infected cells. Log2FC > 0.5 and Log2FC<-0.5, and adjusted *P*-value = 10e-5 used as cutoffs. (B) Venn diagrams represent the overlap between upregulated (right) and downregulated (left) genes in HCV-infected, SARS-CoV-2-infected and HCV-SARS-CoV-2-coinfected cells. The numbers in the green and blue circles refer to percentage of genes from total SARS-CoV-2 and HCV differentially expressed gene set, respectively, located in each area of the circle. At regions where HCV and SARS-CoV-2 sets are overlapped, the green and blue percentages correspond to SARS-CoV-2 and HCV data, respectively. (C) Barplot of differentially enriched pathways, obtained from KEGG database, of HCV (left) and SARS-CoV-2 (right) data. Bars represent the significance level of enrichment in SARS-CoV-2-infected, HCV-infected, and HCV-SARS-CoV-2-coinfected cells (red, green and blue bars, respectively). *P*-value (adjusted) = 0.05 used as cutoff.

To further investigate the interplay between HCV and SARS-CoV-2 coinfection and the host cell, we performed a transcriptome analysis of mono- and coinfected cells. Analysis of the differential peaks compared to non-infected Huh7.5 cells (using a threshold of adjusted *P* < 10e-5, Log2FC > 0.5 and Log2FC<-0.5) identified 2037 differentially expressed genes (DEGs) in Huh7.5^HCV^ cells (868 downregulated and 1169 upregulated), 4335 DEGs in cells infected with SARS-CoV-2 only (1906 downregulated and 2429 upregulated), and 2840 DEGs in HCV-SARS-CoV-2 coinfected Huh7.5 cells (1686 downregulated and 1154 upregulated) (Fig. 7A). Taken together, both viruses, either alone or in coinfection, massively alter the gene expression pattern of the host hepatocyte. Most of the downregulated DEGs in the Huh7.5^HCV^ cells (76%) were also downregulated in the coinfected cells (Fig. 7B). In parallel, a substantial proportion (31%) of the DEGs upregulated in the HCV infection group were also upregulated in the coinfected group (Fig. 7B). Lower preservation was observed for the genes dysregulated upon SARS-CoV-2 infection compared to HCV-SARS-CoV-2 coinfection; of all the SARS-CoV-2 DEGs, 53% were downregulated and 24% were upregulated in the coinfected cells (Fig. 7B). Among the DEGs identified in the coinfected cells, most (75% downregulated and 74% upregulated) were also observed in either HCV/SARS-CoV-2 mono-infected cells or in the coinfected cells, while the remaining DEGs were unique to the coinfection group (Fig. 7B). Among the DEGs, we identified ACE2 as significantly upregulated in the coinfected cells in the RNA-seq data. Altogether, the shared DEGs analysis demonstrates partial preservation of DEGs between the HCV/SARS-CoV-2 mono-infections and HCV-SARS-CoV-2 coinfection, with more shared downregulated than upregulated genes.

**FIG 8:**
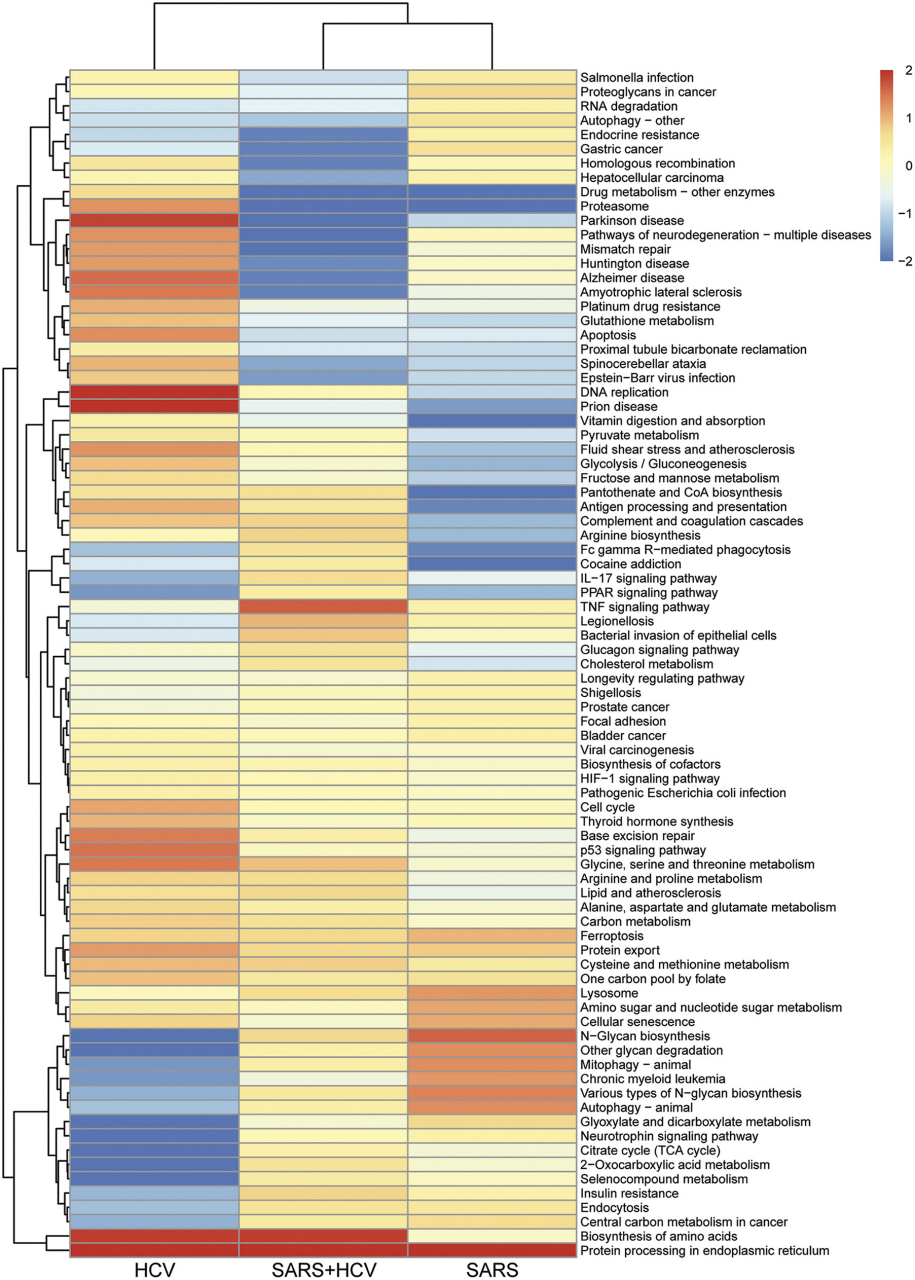
Overall analysis of pathways enriched in at least one of the groups (SARS, HCV, and HCV-SARS-CoV-2). Heatmap of all the pathways enriched in at least one of the infected cell groups (HCV, SARS-CoV-2 and HCV-SARS-CoV-2). The dendrogram was made by hierarchical clustering applied on enrichment levels. Values represent log(-log_10_[adjusted *P*-value]) from pathway analysis. All values below -2 and larger than 2 were set to be -2 and 2, respectively.

A pathway analysis performed to gain deeper insights into the nature of the host environment that supports the replication of the 2 viruses in a coinfection state, found that the top pathways enriched in HCV monoinfection were also enriched to some degree in the coinfected cells (Fig. 7C, left). Similarly, the most enriched pathways in SARS-CoV-2 moninfection were also enriched in coinfected cells (Fig. 7C, right). The heatmap in Fig. 8 presents the pathways that were enriched in at least one of the groups, and shows that many pathways overlapped between the three groups. Interestingly, many of the shared pathways were recently identified in genetic screens and transcriptomic and proteomic analyses as altered by SARS-CoV-2 and were shown to play a role in the viral life cycle and were also implicated in HCV infection and propagation. The most enriched pathway in all 3 groups was “Protein processing in endoplasmic reticulum” (ER) (Fig. 8). Both viruses require a tight interaction with the host cell endomembrane organelles. HCV recruits the ER to form a membranous web for the translation and replication of its RNA genome (44). ER-derived membrane structures are indeed essential for replication, translation, post-translation protein modifications, and assembly of SARS-CoV-2 (45). Enriched pathways also included cholesterol and lipid metabolism (Fig. 8), which is essential for the life cycle of both viruses as well; cellular cholesterol is required for SARS-CoV-2 entry (14) and lipid metabolism is essential throughout its life cycle (46) and is dysregulated in COVID-19 patients (47). Similarly, HCV alters the expression of genes involved in cholesterol and lipid metabolism, resulting in their accumulation, which facilitates HCV replication, assembly, and release (48), and also results in hepatic steatosis in HCV patients (49). Other pathways common to the two viruses and which have been implicated in the life cycle of both viruses, included amino acids metabolism and biosynthesis of amino acids, focal adhesion, HIF-1 signaling pathway, cell cycle, and lysosome (Fig. 8) (50–56). Overall, the transcriptome analysis suggested extensive convergence of host factors dependency of both viruses that are dysregulated also in the coinfected cells, which, most likely, enable efficient co-replication in the host hepatocytes.

## DISCUSSION

Identification of viral coinfections and understanding virus-virus interactions, and their influence on the host cell are emerging fields in virology. It is evident that preinfection with one virus may affect the infection cycle, disease course and outcomes of a second virus. Viral coinfections may result in competition, interference, and replication suppression or enhancement (57). Most of the reported data describe the effect of coinfections of two viruses on the organism level. However, little is known about the interactions between coinfecting viruses at the cellular level (57). Here, we provide the first experimental evidence for the coexistence of SARS-CoV-2 and HCV infections, and the interplay between both viruses. The study revealed a complex relationship of enhancement between the two viruses, where HCV infection increased the expression of the SARS-CoV-2 entry receptor ACE2, thus facilitating SARS-CoV-2 entry, and potentially, HCV entry. Thereafter, SARS-CoV-2 infection enhanced HCV replication in hepatocytes.

Although interference of one virus with the replication of a coinfecting virus via activation of innate immune responses is the most common outcome of coinfections, enhancement as shown here, has also been acknowledged (57). Relationship of enhancement was recently reported in coinfection of SARS-CoV-2 with influenza A virus (IAV) at the organism level. Increased expression of ACE2 triggered by preinfection with influenza A virus (IAV) significantly enhance SARS-CoV-2 infection (58). Increased IAV viral load was also observed in SARS-CoV-2-IAV coinfection (59). In addition, the SARS-CoV-2-IAV coinfection triggered severe disease and higher mortality compared to each infection alone (59). Enhancement of HCV infection was observed both at the organism and cellular levels in coinfections with human immunodeficiency virus (HIV) and hepatitis B virus (HBV). A low level of HIV infection and replication was suggested in hepatocytes (60), and an enhancing effect on HCV life cycle and disease outcome were reported (61). The impact of HIV on HCV infection was suggested to be related to the HIV-mediated acquired immunodeficiency or direct virus-virus and virus-host interactions (61). HIV infection was shown to directly increase HCV replication through enhancement of TGF-1 expression mediated by the HIV protein gp120 (62). Moreover, accumulation of HCV RNA was observed in HIV-HCV coinfected cells and advanced liver fibrosis was observed in HIV–HCV coinfected patients (63). HBV/HCV coinfection in the liver is rather common, with rates ranging between 1% and 15%, and is associated with increased liver disease severity and mortality compared to monoinfections (64–66). Since both viruses primarily infect hepatocytes, direct interaction between the two viruses is possible, despite their entirely different life cycles. *In vitro* experiments suggest impaired HBV replication in HCV-infected hepatocytes, due to IFN signaling or direct virus-virus interactions, while HCV replication is not affected by HBV (66, 67). Other studies suggest efficient co-replication of both viruses without interference (68, 69). Here, since SARS-CoV-2 and HCV are two unrelated viruses, it is less probable that their coexistence in hepatocytes and the enhanced HCV replication in response to SARS-CoV-2 is mediated by trans-complementation of viral proteins that directly facilitate their replication. It is more likely that the effects of these viruses on the host cell is indirect, by creating a supporting environment that has a synergistic effect on the replication of both viruses. Indeed, the changes in the host cell induced by both viruses converge to common signaling pathways that are hijacked by both viruses for their own propagation.

Importantly, we showed enhanced SARS-CoV-2 entry in HCV-preinfected hepatocytes, which was associated with an HCV-mediated increase in ACE2 expression. An intriguing question is whether the increase in ACE2 expression is related to an HCV-specific mechanism or to a cellular antiviral response, which may be triggered in response to any virus that induces innate immune signaling. A recent study reported that ACE2 is an interferon-stimulated gene (ISG) in airway epithelial cells and suggested that the interferon-mediated upregulation of ACE2 expression is exploited by SARS-CoV-2 to enhance infection (70). However, another study demonstrated that not the full, but, rather, a truncated isoform of ACE2 that does not bind SARS-CoV-2 spike protein is an ISG (71), and therefore, induction of IFN by a co-infecting virus is not a likely mechanism of enhanced cellular entry of SARS-CoV-2. In support of this conclusion, an IAV-mediated increase in ACE2 expression was shown to specifically enhance SARS-CoV-2 entry in IAV-preinfected cells, but not in cells infected with a panel of other viruses that cause common cold in humans (58). Importantly, IFNα treatment did not promote SARS-CoV-2 infection, and did not significantly increase ACE2 expression. Moreover, the enhanced ACE2 expression and SARS-CoV-2 infectivity were maintained in IAV-preinfected IFNAR–/– A549 cells lacking the IFN-α receptor (58). In line with these observations, the present work showed that ACE2 expression was not enhanced by replication of subgenomic HCV RNA replicons or HBV, suggesting that the enhanced ACE2 expression in response to infection is virus-specific.

Another important finding is the role of ACE2 in the HCV life cycle. Further investigation is needed to uncover the nature of ACE2-dependent enhancement of HCV entry. Specifically, it remains to be determined whether ACE2 expression levels affect HCV entry via direct interaction between the E1E2 envelope proteins and ACE2, rendering ACE2 an attachment factor, receptor, or co-receptor. Alternatively, the impact of ACE2 on HCV may be indirect.

It is worth noting that most of the experiments in this study were performed using the Huh7.5 cancer cell line due to the limitation of low infection efficiency in primary hepatocytes. Further study is needed to confirm the results in more physiological conditions.

In summary, this study provides the first experimental evidence for SARS-CoV-2-HCV coinfection of liver cells, and suggests that HCV infection is a risk factor for enhanced liver disease in coinfected patients. Although this outcome is supported by recent clinical findings (40–42), the specific effects of enhanced SARS-CoV-2 infection and HCV replication in coinfection states on liver pathology remain to be evaluated in clinical settings. In a broader view, this study emphasized the importance of identifying coinfecting pathogens that increase the risk of SARS-CoV-2 infection and that may accelerate COVID-19-related co-morbidities.

## MATERIALS AND METHODS

### Cell lines.

Huh-7.5 cells (a generous gift from Charles Rice, Rockefeller University), Huh7/FT3-7 cells (a generous gift from Stanley M. Lemon, University of North Carolina at Chapel Hill), HepG2 (ATCC), HEK293 (ATCC), and HEK293 cells stably expressing hACE2 (HEK293-ACE2) (in-house preparation) were cultured in Dulbecco’s modified Eagle’s medium (DMEM) (Gibco), supplemented with 10% fetal bovine serum (FBS), 1% l-glutamine, 1% penicillin-streptavidin, and 1% non-essential amino acids (NEAA). African green monkey kidney clone E6 cells (Vero E6, ATCC CRL-1586) were grown in DMEM containing 10% FBS, MEM NEAA, 2 mM l-glutamine, 100 U/mL penicillin, 0.1 mg/mL streptomycin, and 12.5 U/mL nystatin (P/S/N) (Biological Industries). Normal primary human hepatocytes (PHH) (Gibco) were cultured on collagen (Gibco) in William's Medium E (Gibco), supplemented with cocktail B (Gibco) and HEP-extend (Gibco). The HCV-N NS3-NS5B subgenomic replicon cell line was cultured as we previously described (72). All cells were cultured at 37°C, 5% CO_2_, with 95% humidity.

### Preparation of HCV stock and infection.

HJ3–5 is a H77C/JFH1 chimeric HCV containing core-NS2 proteins from genotype 1a and NS3-NS5B from genotype 2a (a generous gift from Stanley M. Lemon, University of North Carolina at Chapel Hill). Virus stocks were produced in Huh7/FT3-7 cells and viral titers were determined using the focus forming unit (FFU) assay in Huh-7.5 cells, as we described previously (73). For all experiments with 100% HCV-infected Huh7.5, cells were infected with HJ3-5 at a multiplicity of infection (MOI) of 0.1, and passaged for at least 2 weeks until approximately 100% of the cells were HCV-positive, as determined by immunofluorescence, as we previously described (72). PHH cells were infected with HJ3-5 at MOI 1; the HCV inoculum was incubated with PHH for 8 h. Then, hepatocytes were washed and incubated with fresh medium. Cell culture medium was replaced at 24 h intervals. HCV-cured cells were prepared as we previously described (55). In brief, HCV-infected cells (~100% infected, generated as described above) were treated with 0.5 μM sofosbuvir, 0.5 μM daclatasvir, and 7.5 nM paritaprevir for 14 days. Following treatment, cells were immunostained with HCV-positive serum from an infected subject and then with anti-human 488 Alexa flour (Jackson) as secondary antibody to confirm absence of HCV infection. Cured cells were used for experiments 1 month following curing.

### Preparation of SARS-CoV-2 stock and infection.

SARS-CoV-2 (GISAID accession EPI_ISL_406862) was kindly provided by Bundeswehr Institute of Microbiology, Munich, Germany. Virus stocks were propagated (4 passages) and titered on Vero E6 cells. All SARS-CoV-2 virus handling was conducted in a biosafety level 3 facility, in accordance with the biosafety guidelines of the Israel Institute for Biological Research.

For HCV-SARS-CoV-2 coinfection experiments, 100% HCV-infected or non-infected Huh7.5 cells were seeded in 12- or 24-well plates, or on 8-well chamber slides (Thermo) and grown overnight. The next day, SARS-CoV-2 stock was used to infect the cells at an MOI of 0.01 for 1, 5, 24, or 48 h. Then, cells were lysed for RNA purification, or fixed for immunostaining for detection of infection.

### Immunofluorescence staining.

Cells were seeded in 8-well chamber slides (Thermo) and fixated with 3–4% formaldehyde for 15–30 min at room temperature. To detect HCV infection, cells were immunostained with human serum derived from HCV-infected patient, followed by incubation with anti-human Cy3 (Jackson) secondary antibody. To detect SARS-CoV-2 infection, the fixed cells were blocked with PBS containing 2% FBS and stained with hyperimmune rabbit serum from intravenous SARS-CoV-2-infected rabbits (in-house preparation), and then with Alexa Fluor 488-conjugated goat anti-rabbit IgG (Sigma). To detect ACE2 expression, cells were immunostained with mouse anti-ACE2 (Abcam) or goat anti-ACE2 (R &D systems), and then with anti-mouse 488 Alexa fluor IgG (Jackson) and anti-goat IgG (Invitrogen), respectively, and counterstained with DAPI. Images were obtained with a Zen Live Imaging (Time Lapse) microscope (Zeiss) or confocal microscope (LSM 780 + Chameleon Vision II).

### Quantitative real-time RT-PCR.

Total RNA was extracted from cells using a RNA kit (Qiagen). Equal amounts of the RNA isolated from treated or control cells were transcribed into cDNA, using the High-Capacity cDNA Reverse Transcriptase kit (Applied Biosystems) and analyzed by RT-PCR using the Power SYBR green Master Mix (Life Technologies). The thermal program included 40 cycles at 95˚ for 20 s and 60˚ for 20 s. Differential expression was calculated using the equation of 2^(-ΔΔCt), with GAPDH as an endogenous control. RT-PCR analysis was conducted using RNA from 3 independent experiments.

Real-time quantitative RT-PCR was carried out using primers for: the 3-untranslated region (UTR) of HCV (FW GCCATGGCGTTAGTATGAGTGT; REV CCCTATCAGGCAGTACCACAA), ACE2 (FW AGGACACTGAGCTCGCTTCT; REV GAACTTGGGTTGGGCGCTAT), TMPRSS2 (FW AGGTGAAAGCGGGTGTGAGG; REV ATAGCTGGTGGTGACCCTGAG), SARS-CoV-2 spike (FW CAGATGCTGGCTTCATCAAA; REV GGTTGGCAATCAATTTTTGG) and HBV (FW CCACCGTGAACGCCCATC; REV TTGTGCCTACAGCCTCCTAATAC).

### Preparation of SARS-CoV-2-spike pseudoparticles and transduction experiments.

SARS-CoV-2-spike pseudoparticles were generated by co-transfection of Expi293F cells with pCMV delta R8.2, pLenti-GFP (Genecopoeia), and pCDNA3.1 spikeΔC19 according to manufacturer’s instructions (ThermoFisher Scientific) at a ratio of 1:2:1, respectively. The supernatant was harvested 72 h posttransfection, centrifuged at 1500 × *g* for 10 min to remove cell debris, and passed through a 0.45 μm filter (LIFEGENE, Israel). Next, the pseudoparticles-containing supernatant were concentrated to 5% of their original volume using Amicon Ultra Centrifugal Filter Unit with 100 kDa cutoff, at 16°C (Merck Millipore).

HCV-infected and non-infected cells were seeded in 96-well plates (Greiner) or 8-well chamber slides (Thermo). The following day, concentrated pseudoparticles were added in the pre-seeded wells and incubated for 48 h. Thereafter, the medium was replaced with fresh DMEM without Phenol Red, and the plates were imaged 24 h later with the IncuCyte ZOOM system (Essen BioScience), under a 10× objective. Default IncuCyte software settings were used to calculate number of GFP-positive cells in four GFP-channel images from each well.

To inhibit infection, HCV-infected and non-infected cells were seeded on 8-well chamber slides (Thermo). Cells were incubated with 5 mg/mL anti-ACE2 antibody (R &D systems) for 1 h, and then overnight with pseudoparticles. Images of the cells were obtained with a Zen Live Imaging (Time Lapse) microscope (Zeiss) and quantified using Image Pro 10 software.

### Transfections.

Huh7.5 cells were plated in a 24-well plate, 18 to 24 h prior to transfection. Fresh culture medium was added to each well 1 h before transfection. Thereafter, the cells were transfected with 0.5 μg pcDNA3.1+/C-(K)DYK-ACE2 (GenScript) diluted in 25 μL serum-free DMEM, or mock transfected, and PolyJet reagent (SignaGen Laboratories), according to the manufacturer’s instructions. Medium was replaced after 6 h and 48 h posttransfection, both transfected and non-transfected cells were infected with HCV at an MOI of 0.1. Cells were harvested 72 h postinfection for RNA isolation.

For knockdown experiments, Huh7.5 cells were seeded in a 24-well plate and incubated until they reached 80% confluence, which was generally 24 h after seeding. Cells were then transfected with ON-TARGETplus Human ACE2 siRNA (Dharmacon) or Non-targeting Pool (Dharmacon) using RNAiMAX (Invitrogen), incubated for 24 h and then infected with HCV at an MOI of 0.1. Cells were harvested 72 h postinfection for RNA isolation.

### Expression and purification of the spike receptor binding domain.

The receptor binding domain (RBD) (residues 319–541) of SASR-CoV-2 were subcloned into a pMT-BiP-His vector (Invitrogen) between the BamHI and NotI restriction sites, downstream to the BiP signal peptide and in frame with a C-terminal 2×StrepTag, cleavable by HRV 3C protease. The plasmid (pMT-RBD) was then co-transfected into S2 Drosophila cells with a puromycin resistance plasmid (pCo-Puro) at a 20:1 ratio, respectively. Cells were selected using puromycin until a stable cell line was obtained.

S2 cells stably expressing the SARS-CoV-2 RBD were grown to in ESF921 (Expression Systems) serum-free medium, at 27° C. When cells reached a density of 1 × 10^7^ cells/mL, protein expression was induced with 600 μM CuSO_4_. Seven days post-induction, cells were pelleted by centrifugation (500 × *g*/4°C/10 min). The medium was further clarified by centrifugation (14,000 × *g*/4°C/10 min), concentrated and then exchanged for 20 mM Tris pH 8.0, 150 mM NaCl using a tangential flow filtration (TFF) system (PALL). The concentrated sample was then loaded onto a StrepTactin column (GE Healthcare), which was then treated following the manufacturer protocols. Elution fractions were analyzed by SDS-PAGE, pooled, and placed on a size exclusion chromatography column (Superdex200, GE health care) pre-equilibrated with 20 mM Tris pH 8.0, 150 mM NaCl. Relevant fractions were concentrated to 2 mg/mL, aliquoted, flash-frozen in liquid nitrogen, and stored at −80°C until use.

### Flow cytometry.

To evaluate ACE2-RBD binding, 100% HCV-infected Huh7.5 cells, non-infected Huh7.5 cells, HEK293 and HEK293-ACE2 cells, were harvested and fixated with 4% paraformaldehyde. The fixed cells were then incubated for 1 h at room temperature with 1 μg/mL purified recombinant RBD diluted in 2.5% BSA. Cells were washed and then incubated for 1 h at room temperature with 555 fluorophore-conjugated anti-RBD antibody (Sino biological). Flow cytometry analysis was performed using a Gallios Flow Cytometer (Beckman Coulter) and FlowJo software (BD). To evaluate cell surface level of ACE2, the cells were treated as above, but incubated with rabbit anti-ACE2 antibody (Abcam) followed by 488 conjugated goat anti-rabbit secondary antibody.

### Cell-cell fusion assay.

All 100% HCV-infected or non-infected Huh7.5 cells were seeded in 24-well plates. The next day, cells were transfected with pCDNA3.1 spikeΔC19 using PolyJet (Signagen Laboratories), according to manufacturer protocol. After a 4-day incubation, CellMask (Thermo-Fisher) membrane dye was applied according to the manufacturer’s instructions. Images were taken using a Zen Live Imaging (Time Lapse) microscope (Zeiss), and then cells were fixated with 4% paraformaldehyde and stained with DAPI. The fusion index was calculated by measuring the ratio between percent of area stained with DAPI (nuclear stain) and the percent of area stained with membrane dye using ImageJ.

### Preparation of HBV lentiviruses and transduction experiment.

HEK293T cells were seeded in 9 cm dishes and, the next day, were transfected with the lenti expression vector, PHR- HBV or PHR-GFP packaging vector (cytomegalovirus delta‐R8.9), and VSV‐G vector, using PEI (Sigma). Growth medium was replaced after 8 to 16 h and lentivirus-containing supernatant was harvested 24 and 48 h later.

For HBV lentivirus infection, HepG2 cells at 50% – 70% confluence were transduced. The following day, viral medium was added to the cells with 8 μg/mL Polybrene (Sigma-Aldrich). After 24 h, the cells were washed with PBS and used for experiments.

### Western blot.

Total cell lysates were separated by SDS-PAGE. The proteins were transferred to nitrocellulose membranes and incubated overnight with primary antibodies specific to ACE2 (Abcam), HCV Core (Invitrogene), and β actin (Millipore) and then with HRP-conjugated secondary antibody for 1 h. Reactive bands were visualized with ECL reagent (Pierce) using the Platinum Q9 gel documentation system. Protein levels were quantified and normalized to β actin levels using Image Studio Lite software.

### Analysis of ACE2 expression in liver biopsies.

RNA-seq data were obtained from the NCBI Gene Expression Omnibus (GSE84346) using the R statistical software (www.r-project.org). The data set included gene expression of liver biopsies from normal and HCV-infected patients. HCV-infected patients was sub-classified into low interferon-stimulated genes and high interferon-stimulated genes, and only patients with low interferon-stimulated genes were used for the analysis. Student's *t* test was used to compare the distributions of ACE2 expression.

### Preparation of HCV pseudoparticles (HCVpp) and transduction experiments.

HCVpp were generated by transfecting HEK293T cells with a plasmid encoding the HCV E1E2 gene (a generous gift from Prof. Mansun Law, The Scripps Research Institute), using PEI. Twenty-four hours after transfection, VSVΔG-G viruses (containing a GFP-reporter) were added to the cell cultures. Six hours later, plates were washed 6 times with PBSX1, and fresh medium was added. After 48 h, the supernatant was collected and HCVpp were concentrated x40 using Amicon Ultra with 100 kDa cutoff. Huh7.5 cells were seeded in 24-well plates and incubated for 24 h. Cells were then transfected with a plasmid encoding the ACE2 gene (GenScript), using Lipofectamine 3000 (Invitrogen) according to the manufacturer’s instructions. Two days after transfection, concentrated HCVpp were added to the wells and the GFP-positive cells were monitored and counted using the IncuCyte ZOOM system (Essen BioScience).

### Assessment of HCV binding to hepatocytes.

Huh 7.5 cells were seeded 24 h before transfection to reach 90% confluence at the time of transfection. Cells were then transfected with a plasmid encoding the ACE2 gene (GenScript), using Lipofectamine 3000 (Invitrogen) according to manufacturer protocol. The cells were collected 48 h after transfection, and incubated with HCV at an MOI of 1 for 1 h, at 4°C. Thereafter, they were gently washed 3 times with PBS to remove unbound virus, harvested, RNA was isolated, and qRT-PCR was performed with HCV-specific primers.

### RNA-seq analysis.

We performed RNA-seq for Huh7.5HCV cells (100% HCV-infected), Huh7.5 cells infected with SARS-CoV-2 for 48 h, Huh7.5HCV cells coinfected with SARS-CoV-2 for 48 h, and non-infected Huh7.5 cells as control.

### (i) Library preparation.

Total RNA from cells (generated as described above) was purified using the RNeasy minikit (Qiagene). Next, 1 μg of total RNA was treated with the NEBNext poly (A) mRNA Magnetic Isolation Module (NEB). RNA-seq libraries were produced using the NEBNext Ultra RNA Library Prep Kit for Illumina (NEB). For each tested group 3 biological replicates were performed. All libraries were sequenced by Illumina MiSeq platform with single-end reads of 50 bp, according to the manufacturer’s instructions.

### (ii) Sequence alignment.

RNA-seq analysis was performed on data from SARS-CoV-2-infected cells (*n* = 3) and HCV+SARS-CoV-2 coinfected cells (*n* = 3) obtained in this study, and on data from HCV-infected cells (*n* = 6) and uninfected cells obtained from Perez et al. (55). Raw reads were aligned to the NCBI RefSeq genes from the GRCh37/hg19 human genome assembly using RNA-seq by expectation maximization pipeline (RSEM) (74).

### (iii) Differential expression analysis.

Differential gene expression analysis was conducted using the DESeq2 (75) package (release 1.30.1) in R (version 4.0.5). Specifically, data from HCV-infected, SARS-CoV-2-infected, and HCV-SARS-CoV-2-coinfected cells were compared separately to data from non-infected cells, using *P*-value (adjusted by Benjamini–Hochberg method) of 10e-5 and the absolute fold change of 0.5 as cutoffs. To avoid biases resulting from different number of replicates, we iteratively sampled 3 out of 6 samples for the HCV and non-infected categories, and used the average value over iterations.

### (iv) Pathway enrichment analysis.

Pathway analysis of differentially expressed genes (both up- and downregulated genes) was performed with ‘KEGGprofile’ R package (release 1.32.0) using the find_enriched_pathway function with the following parameters: returned_adjpvalue = 0.05, download_latest = T.

### (v) Data visualization.

Volcano plots of differential gene expression were generated using the ‘EnhancedVolcano’ package (release 1.8.0) in R. Venn diagrams visualized intersection of differentially expressed genes between HCV, SARS-CoV-2, and SARS-CoV-2+HCV groups ‘BioVenn’ R package (release 1.1.3).

Barplots were prepared to present the significance of differentially enriched pathways for the different sets, using ggplot2 R package (release 3.3.5).

A heatmap was prepared using ‘pheatmap’ R package (release 1.0.12), to visualize the levels of pathway enrichment for different sets. The dendrograms accompanying the map was prepared by a built-in hierarchical clustering approach. For the heatmap, enrichment level was set to be transformed, such as log(-log_10_[adjusted *P*-value]), adjusted *P*-values from the pathway analysis. All the values below -2 and greater than 2 were replaced by -2 and 2, respectively.

### Data availability.

The RNA-seq data generated during the current study have been deposited in GEO repository with accession code GSE207151.
